# Improvement in Insulin Sensitivity Prevents Decline in Glucose Uptake, Functional Connectivity, and Volume in the Insulin Resistant Human Brain

**DOI:** 10.21203/rs.3.rs-7462946/v1

**Published:** 2025-09-09

**Authors:** K. Sreekumaran Nair, Mark Pataky, Gregory Ruegsegger, Hang Joon Jo, Katherine Klaus, Kyle Sevits, Robert Leija, Arathi Kumar, Seokbeen Lim, Sumaiya Sinhawansa, Martina Dreconga, Claudio Cobelli, Rickey Carter, Nikki Stricker, Val Lowe, John Port

**Affiliations:** Mayo Clinic; Mayo Clinic; Mayo Clinic; Hanyang University; Mayo Clinic; Mayo Clinic; Mayo Clinic; Mayo Clinic; Mayo Clinic; Mayo Clinic; University of Padova; University of Padova; Mayo Clinic; Mayo Clinic; Mayo Clinic; Mayo Clinic

## Abstract

Insulin resistance (IR) is a modifiable risk factor for dementia, yet its effects on brain metabolism and function remain unclear. In older adults, greater IR was associated with reduced cerebral glucose uptake (indicating impaired mitochondrial metabolism), atrophy, and weakened connectivity between brain regions critical for cognition. In neuron-specific insulin receptor knockout mice, brain IR produced deficits in hippocampal- and prefrontal-dependent tasks accompanied by reduced brain mitochondrial ATP and elevated reactive oxygen species. To evaluate reversibility of IR-induced brain deficits, forty older adults with IR were randomized to 40-weeks of metformin or placebo. Metformin improved insulin sensitivity, increased brain glucose uptake, strengthened cognitive network connectivity, and preserved whole-brain and regional volumes implicated in decision-making and learning. Metformin also improved processing speed and working memory. Collectively, these findings highlight IR as a driver of brain metabolism and support the concept that insulin sensitization can prevent neurobiological deficits in older people with IR.

## INTRODUCTION

With the expanding elderly population, the prevalence of dementia is surging with profound socio-economic consequences. In the U.S., the estimated lifetime risk of dementia after age 55 is 42%^1^. Lifetime dementia risk is even higher – ranging from 45% to 60% – among women, black adults and APOE ε4 carriers. Notably, conditions with insulin resistance (IR) such as T2DM, obesity, pre-diabetes and metabolic syndrome are associated with a 1.5-to-4-fold increased risk of Alzheimer’s Disease (AD) and other dementias^2, 3^. The total annual cost of caring for individuals with dementia in the U.S. is estimated to be around $393 billion in 2025 which is predicted to exceed $1 trillion by 2045^4^. Dementia linked to IR is expected to rise sharply, driven by increasing rates of obesity^5^, prediabetes^6^ and T2DM^7^. There is a critical need for a greater understanding of pathophysiology and advancing therapies to prevent and treat dementia in these vulnerable populations with IR.

An important question is why people with IR are at elevated risk for dementia. Insulin, a critical regulator of skeletal muscle energy metabolism, facilitates uptake of glucose, fatty acids, and amino acids, and regulates mitochondrial conversion of these fuels into ATP - the chemical energy needed for all cellular functions. Insulin deficiency or resistance impairs mitochondrial function^8, 9, 10, 11^, thus reducing ATP production efficiency and increasing oxidative stress in muscle. While skeletal muscle constitutes ~40% of body weight, it accounts for ~20–25% of resting energy expenditure (REE). In contrast, the brain comprises only ~2% of body weight yet consumes ~20% of REE, making it the body’s most energy-demanding organ^12^. Preclinical studies have demonstrated that insulin receptors are rich in brain regions intimately involved in cognition such as the cerebral cortex and hippocampus^13, 14, 15, 16, 17, 18^. ATP availability is indispensable for neuronal connectivity, neurotransmission, and signaling cascades supporting key cognitive functions^19^.

In rodent models, insulin enhances mitochondrial biogenesis and oxidative phosphorylation in brain regions rich in insulin receptors such as the hippocampus and cerebral cortex, effects which can be inhibited by insulin-receptor antagonists^20, 21^. Moreover, high-fat-diet induced IR adversely affects brain regions critical for cognition by uncoupling oxidative phosphorylation, thus diminishing ATP production and increasing ROS emission, thereby inducing oxidative damage^22^. In IR mice, interventions that improve insulin sensitivity, such as aerobic exercise or metformin, mitigate these impairments, suggesting enhanced insulin action supports brain ATP availability, which is essential for functional connectivity and communication between brain regions^22^. Aerobic exercise training has been shown to improve brain glucose uptake in both young and older people^23^ with potential improvement in cognitive functions^24^.

Metformin is the most prescribed insulin sensitizer for T2DM and tantalizing data suggests its benefits on cognition and brain metabolism^25, 26, 27^. In prior work, we demonstrated that metformin improved brain mitochondrial function in IR mice, enhancing ATP production and reducing ROS emission^22^. However, other studies, particularly those using AD models, have reported mixed or adverse effects of metformin on cognitive outcomes^28, 29^. Observational studies in T2DM suggest that metformin slows or reduces the prevalence of dementias^30^ and metformin cessation may increase dementia risk^31^. However, metformin treatment is reported to result in Vitamin B12 deficiency^32^, which may contribute to dementia^33^, potentially explaining the conflicting results. However, it remains to be determined whether metformin administration in a prospective study increases glucose uptake in brain and causes associated changes in brain regional connectivity and improves brain volume.

Brain mitochondrial energy metabolism can be represented by brain glucose uptake measured by ^18^F-FDG PET^34^, as glucose is the obligatory brain fuel for ATP production. We hypothesized that reduced brain glucose uptake occurs in an IR state concomitant with reduced connectivity between different brain regions, as measured by fMRI. We also measured regional brain volumes by volumetric MRI to determine whether reduced regional brain volumes occur in IR, as reported^35, 36^. We also created an inducible neuron-specific insulin-receptor deficient experimental mouse model to establish whether inducing brain IR reduces mitochondrial ATP production rate concurrent to cognitive defects. Importantly, in a randomized, double-blind, placebo-controlled study in 40 people with IR (>60 years of age), we determined whether enhancing insulin sensitivity with metformin administration improved brain metabolism by increased glucose uptake concurrent to improvement in whole brain and regional volumes, connectivity and cognition. In the current study, all participants received Vitamin B12 supplementation, and we monitored circulating levels of both metformin and Vitamin B12. Our findings provide substantial evidence supporting metformin’s cognitive and metabolic benefits in older adults with IR.

## RESULTS

### People with Whole Body Insulin Resistance Display Lower Glucose Uptake in Brain Regions Critical for Executive Function, Sensory Integration, and Attention.

**Figure 1:**
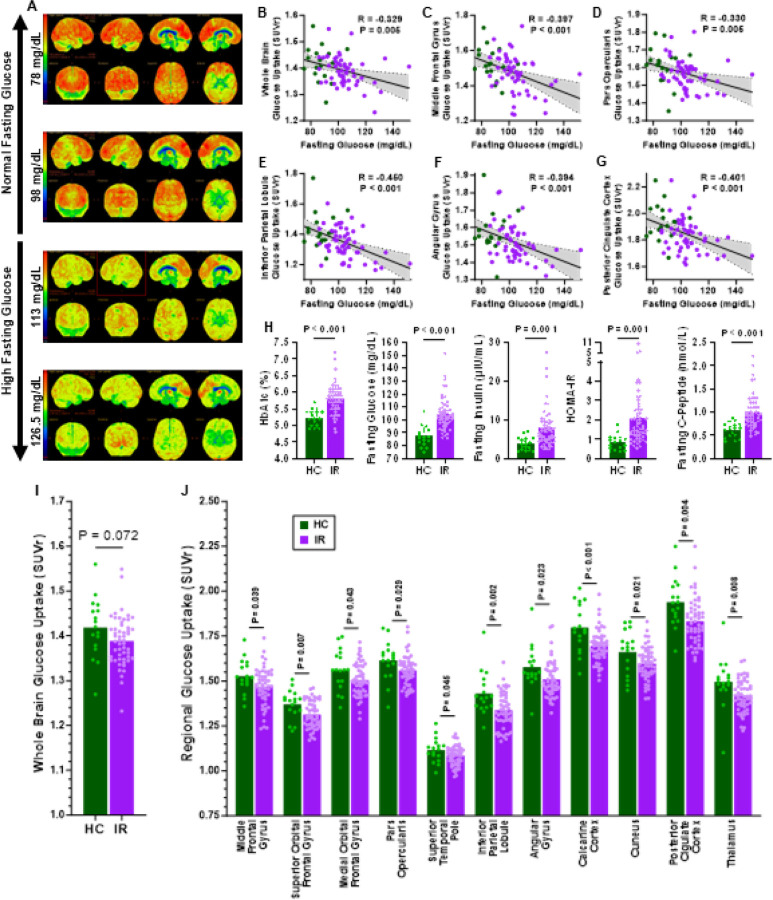
People with Whole Body Insulin Resistance Display Lower Glucose Uptake in Brain Regions Critical for Executive Function, Sensory Integration, and Attention. (**A**) Standardized 3-dimensional FDG SSP (Stereotactic Surface Projection) intensity maps demonstrate whole brain glucose uptake across ranging levels of fasting glucose. (**B-G**) X-Y plot demonstrate the significant negative correlation between fasting glucose and glucose uptake in the whole brain, middle frontal gyrus, pars opercularis, inferior parietal lobule, angular gyrus, and posterior cingulate cortex. Sample size contributing to linear regression analysis between brain glucose uptake and fasting plasma glucose was n=70 (Healthy Control, HC=17, green dots; Insulin Resistant, IR=53, purple dots). (**H**) Group differences in HbA1c, controlled fasting glucose, insulin, C-peptide, and HOMA-IR. Sample sizes were n=18 for HC and n=56 for IR for all outcomes except for controlled fasting C-peptide (IR=55) and HbA1c (IR=54). (**I-J**) Glucose uptake in the whole brain (**I**) and in different brain regions (**J**) measured by ^18^FDG-PET and displayed as relative standardized uptake values (SUVr) (HC, n=17; IR, n=55). Dots represent individual participants. Simple linear regression analysis was used for panels in B-G. Two-sided Student’s t-tests were used to compare group differences for panels in H-J.

Firstly, we determined whether markers of reduced insulin action are determinants of brain glucose uptake. As glucose is the brain’s obligatory fuel for ATP production, reduced uptake indicates impaired mitochondrial metabolism. Here, we demonstrate in a cohort of 74 adults (age 50–79 years), including both insulin sensitive people and people with IR, that glucose uptake across the whole brain is negatively associated with fasting glucose (Figures 1A–B). Fasting glucose was also negatively associated with glucose uptake in specific anatomical regions (Figure S1A) including the middle frontal gyrus (Figure 1C), which is involved in attention, working memory, and language-related processing^37^, the pars opercularis (Figure 1D), which is involved in speech production and motor planning^38^, the inferior parietal region (Figure 1E), which integrates sensory information and plays a significant role in attention, language, and social cognition^39^, the angular gyrus (Figure 1F), which is involved in language processing, spatial cognition, and memory retrieval^40^, and the posterior cingular cortex (Figure 1G), which is involved in memory, navigation, and self-referential thought^41^. Fasting glucose is a sensitive biomarker of insulin sensitivity, and its negative association with brain glucose uptake suggests that systemic IR is associated with reduced energy production critical for the important functions in the regions referenced above.

We performed a cross-sectional analysis between people with IR (IR, n=56) and insulin-sensitive healthy controls (HC, n=18). These analyses were drawn from three independent study cohorts (Figure S1B) which were used to reveal correlations between fasting glucose and regional brain glucose uptake (Figure 1A–G). Cohorts were categorized as IR or HC according to established BMI and fasting-glucose thresholds at screening (Figure 1B). Cohorts were of similar age (Figure S1B) and sex distribution (HC = 9 men, 9 women; IR = 26 men, 30 women). The IR group had significantly higher HbA1c (Figure 1H), blood pressure, body fat percentage, and altered blood lipid profile (Figure S1C) versus the HC group. Since the fasting blood draw at the screening visit was based on self-reported length of overnight fast and level of physical activity, a more tightly controlled fasting blood collection was obtained on a separate study visit. For this visit, participants consumed 3 days of standardized weighted meals based on the Harris-Benedict equation and then reported for a supervised overnight stay enabling researcher confirmation of sedentary behavior and length of overnight fasting prior to blood sample collection. Following the controlled overnight stay, the IR group had higher fasting blood glucose, insulin, C-peptide, and HOMA-IR (Figure 1H). These results in people with pre-diabetes are consistent with a previous report showing brain hypometabolism in individuals with T2DM and non-diabetic individuals with poor glycemic control in AD signature regions^42^. Of interest, a subgroup analyses in the above report showed stronger associations of diabetes with hypometabolism for APOE e4 noncarriers, younger subjects, and men suggesting that lower insulin action is more responsible for reduced glucose uptake than susceptibility to AD. The current study demonstrates a clear difference in brain glucose uptake between insulin sensitive people and people with IR, in addition to an association with markers of glycemic status.

Insulin, is traditionally recognized for its role in systemic glucose homeostasis, but it’s role in the central nervous system, influencing neuronal metabolism, synaptic plasticity, and higher-order cognitive functions such as learning and memory^43^, is less understood. Most brain glucose uptake, based on MR Spectroscopy^44^, is independent of insulin. Furthermore, arterio-venous glucose balance studies also have shown that overall brain glucose uptake is not insulin dose-dependent^44, 45^. However, insulin receptors are widely expressed in the human brain^13, 14, 15, 16, 17, 18^. Here, although whole-brain glucose uptake only trended (P=0.072) to be lower in individuals with systemic IR compared to healthy controls (Figure 1I), glucose uptake was significantly (P<0.05) lower in specific brain regions of the prefrontal cortex (middle frontal gyrus, superior orbitofrontal gyrus, medial orbitofrontal gyrus, and pars opercularis), regions of the temporal, parietal, and occipital lobes, the posterior cingulate cortex, and thalamus (Figures 1J and S1D), further supporting the concept that systemic glucose concentrations are inversely related to brain glucose uptake in regions rich in insulin receptors. Although no group differences in cognitive composite scores were observed (Figure S1E), likely due to the large sample sizes typically required to detect such differences in cognition^46^, most regional glucose uptake values trended positively—though not significantly—with processing speed & working memory, and fluid composite cognition scores (Figure S1F), consistent with a previous finding of association between brain glucose uptake and cognition^47^.

### Brain Regions Important for Executive Function and Memory have Fewer Positive Functional Connections in People with Insulin Resistance.

**Figure 2:**
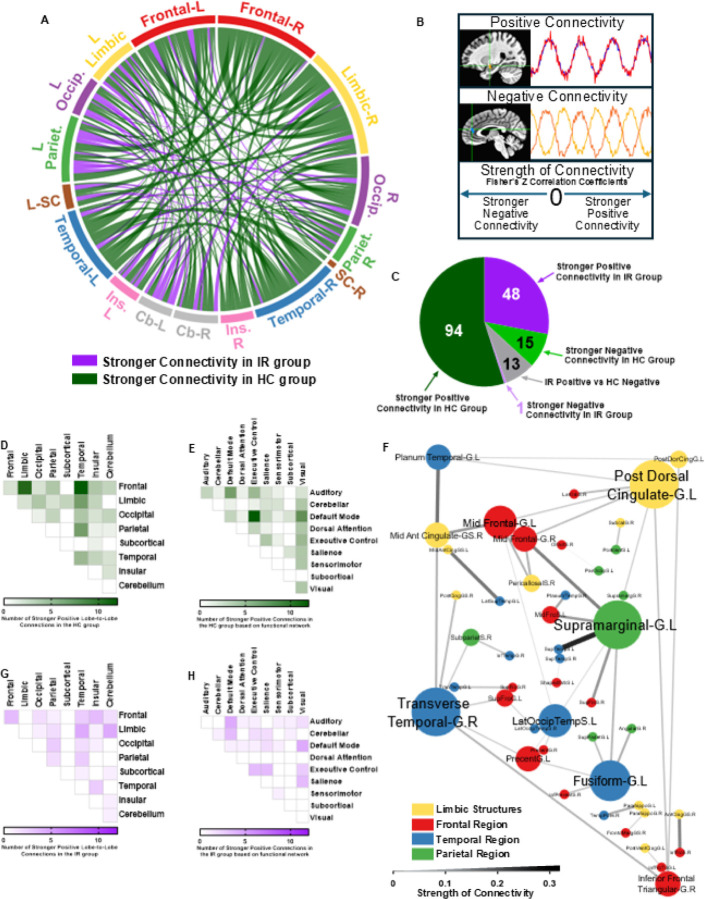
Brain Regions Important for Executive Function and Memory have Fewer Positive Functional Connections in People with Insulin Resistance. (**A**) Chord diagram represents all significant (FDR<0.05) between-group differences (Healthy Control vs Insulin Resistant; HC vs IR) in resting-state functional connectivity measured by fMRI (n=18 HC, and n=56 IR), arranged by hemisphere and lobe. Stronger functional connection in HC (109 connections) and IR (49 connections) is shown as green and purple ribbons, respectively. Ribbon width corresponds to the magnitude of group differences in functional connectivity, computed as the Fisher z-transformed correlation difference (Δz) and weighted by statistical significance (αΔz). Occip. = Occipital, Pariet. = Parietal, SC=Subcortical, Ins. = Insula, and Cb = Cerebellum. (**B**) Time-series examples show positive (in-phase) and negative (out-of-phase) connectivity (correlations), and the strength of connectivity was determined by how far the Fisher z-transformed correlation coefficient was from 0. (**C**) Most of the 171 observed group differences in connectivity were in the positive direction, with stronger positive connectivity in HC (dark green) accounting for 55% of group differences. Stronger positive connectivity in IR (dark purple) accounted for 28% of group differences. (**D**) Stronger positive Frontal-Limbic and Frontal-Temporal connectivity was most prominent in the HC vs IR groups. (**E**) The stronger positive connections in the HC vs IR group were associated with connectivity between the Executive Control and Default Mode networks. (**F**) A functional connection hub map demonstrates the specific frontal, limbic, temporal, and parietal region-to-region connections with stronger positive relationships that accounted for the majority of the HC vs IR group differences. Line width and color demonstrate the strength of connection (based on Fisher’s Z Correlation Coefficients), text and node size increase as the number of connections increase. (**G-H**) Stronger positive connections in the IR vs HC groups were more diffusely distributed across lobes and networks.

Connectivity between different brain regions is important for cognition^48, 49^. We recently demonstrated that transient insulin deprivation in people with type 1 diabetes reduces functional connectivity, especially in hippocampal and cortical regions, corresponding with impaired executive function, attention, working memory, and fine motor speed^50^. To evaluate how chronically reduced insulin action in the IR state impacts functional connectivity, we compared resting-state fMRI between HC and IR groups. We observed 109 stronger region-to-region functional connections in the HC group compared to 49 in the IR group (Figure 2A). Functional connection between two regions can be defined based on either their synchronous activity (“positive connection”) or reciprocal activity (“negative connection”) (Figure 2B). Therefore, we assessed the directionality of functional connections and found that positive connections were mostly responsible for the observed group differences (Figure 2C). The stronger positive functional connections in the HC versus IR group were primarily Frontal-Limbic and Frontal-Temporal (Figure 2D), which corresponded to greater connection between executive control and default mode networks (Figure 2E). Mapping of these specific functional connections together with the third and fourth most represented regions (Temporal-Limbic and Temporal-Parietal) revealed that the posterior dorsal cingulate, middle frontal, and the supramarginal cortices were interconnected and responsible for the strongest and some of the greatest number of connections between HC and IR (Figure 2F). These regions are involved in diverse and higher-order cognitive processes. The posterior dorsal cingulate is a core region in the default mode network, choreographing internally directed cognition and the focus of attention^51^. The frontal lobe, particularly the dorsolateral prefrontal cortex (which includes the middle frontal cortex), is essential for executive functions^52, 53^. The left supramarginal gyrus is critical for is verbal working memory^54^. Together, these regions form a core network supporting higher-order cognitive processes, and disruptions in their connectivity have been associated with altered default mode network activity in individuals with T2DM^55^ and in postmenopausal women at risk for AD^56^. Whereas the stronger positive functional connections that were observed in HC could be localized to specific lobes and networks, the greater positive functional connections in the IR group were distributed across many lobes and networks (Figures 2G–H).

### Insulin Resistance is Related to Lower Brain Volume.

**Figure 3:**
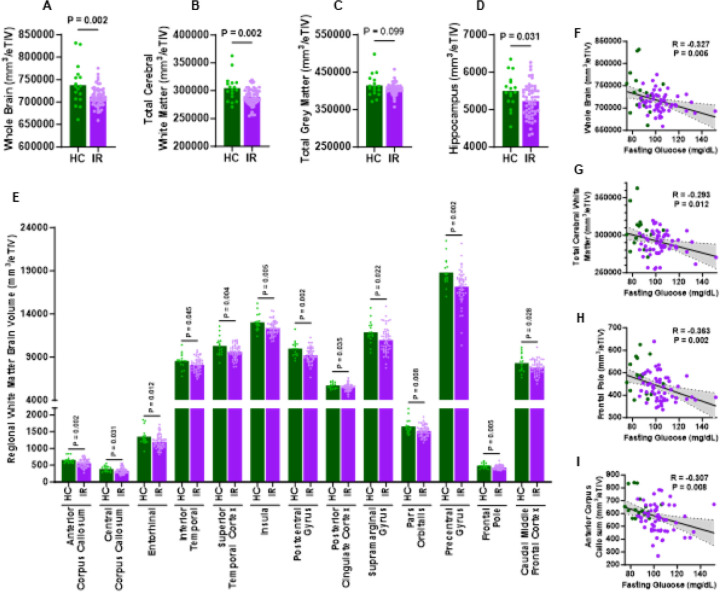
Insulin Resistance is Related to Lower Brain Volume. (**A**) Whole brain (excluding ventricles) (**B**) total cerebral white matter, (**C**) total grey matter, and (**D**) and hippocampal volumes (normalized to estimated intracranial volume, eTIV) in Healthy Controls (HC; n=17; green) and Insulin Resistant (IR; n=56; purple) participants are displayed as mean (bars) with participants represented as individual dots. (**E**) Regional white matter brain volumes that were significantly different (P<0.05) between HC and IR are displayed. Two-sided Student’s t-tests were used to compare group differences. (**F-I**) X-Y plots demonstrate the significant negative correlation between fasting glucose and total brain volume (**F**), total cerebral white matter volume (**G**), frontal pole white matter volume (**H**), and anterior corpus callosum volume (**I**). Dots represent individual participants.

Growing evidence suggests that IR contributes to structural brain deterioration with aging, particularly in white matter and cortical regions involved in memory, executive function, and global cognition^35, 57^. To assess structural effects of IR, we compared total and regional brain volumes between IR and HC groups using volumetric MRI. Brain volumes were normalized to total estimated intracranial volume (eTIV), which eliminated sex-based differences in brain volume^58^ (Figures S2A-F). Total brain volume (excluding ventricles) and total white matter volume were significantly lower in IR versus HC (Figures 3A–B, S2E, and S2G). Although total grey matter volume did not differ between groups (Figures 3C and S2H), the hippocampus was significantly smaller in the IR group (Figure 3D and S2I) and was the only grey matter region significantly different between groups. The hippocampus is essential for forming long-term and episodic memories, spatial navigation, and flexible cognition, and works with the amygdala in emotional memory processing^59^. Our analysis also identified multiple white matter structures with significantly lower volumes in the IR versus HC groups (Figure 3E and Supplementary Figures 2J–V). Fasting glucose was negatively associated with whole brain volume and total cerebral white matter (Figures 3F–G and S2W). Notably, frontal pole volume, a region fundamental for executive control^60^, was negatively associated with fasting glucose (Figures 3H). In addition, fasting glucose was negatively associated with anterior corpus callosum volume (Figures 3I), which connects prefrontal and premotor cortices across hemispheres^61^. The anterior callosal fibers transfer motor information between the frontal lobes^62^. These data indicate that impaired metabolic function during IR reflects early microstructural vulnerability in the white matter regions critical for integrating information and maintaining efficient, coordinated cognitive processes involved in executive function and memory. Corpus callosum volume, which is the largest white matter region in the brain, is related to executive function, attention, and processing speed^60^. We found that total grey matter volume was significantly associated with total composite cognitive score (Figure S2X), and that volumes of all corpus callosum regions, though not statistically significant, showed a general positive association with processing speed & working memory composite score (Figure S2X). Thus, atrophy of white matter regions in combination with hippocampal loss in early stages of IR (even before the onset of T2DM) may initiate the well-documented decline in cognition that occurs with metabolic dysfunction^63^.

### Loss of Brain Insulin Receptor Impairs Brain Mitochondrial Function and Memory in Mice.

**Figure 4:**
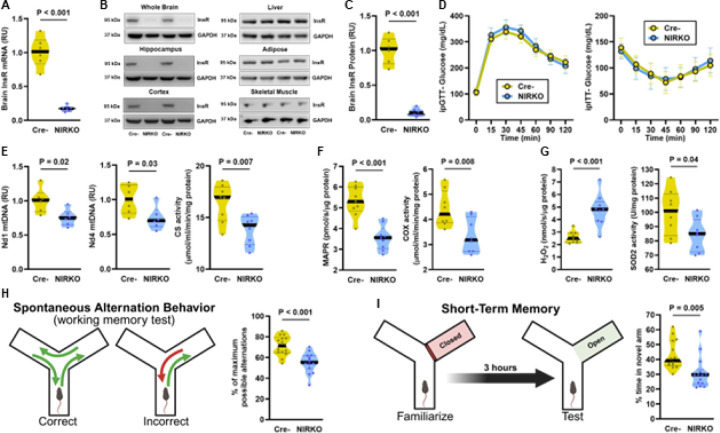
Loss of Brain Insulin Receptor Impairs Brain Mitochondrial Function and Memory in Mice. (**A**) Mice with floxed insulin receptor (InsR) driven by the Nestin promoter what were negative (Cre-) or positive carriers of the CreERT2 gene (NIRKO; Neuron Insulin Receptor Knockout) were injected with tamoxifen (to induce brain InsR knockout in the NIRKO group) and brains were harvested 4 weeks later for quantification of cerebral InsR mRNA by qPCR (n=6 per group). (**B**) Representative immunoblots of the insulin receptor protein across multiple tissues demonstrate that reduction in insulin receptor protein with tamoxifen in the NIRKO mice was specific to the brain. (**C**) InsR protein was quantified in the whole brain (n=6 mice per group). (**D**) Blood glucose values during a fasted (11hr overnight) intraperitoneal glucose tolerance test (ipGTT; 2g/kg of 20% D-glucose injected) and a fasted (6hr) intraperitoneal insulin tolerance test (ipITT; 0.75U/kg insulin injected) 4 weeks following tamoxifen treatment (n=10 per group). Lines graphs presented are presented as mean ± SD. (**E**) Cerebral mitochondrial DNA copy number (Nd1 and Nd4; n=6 per group) and citrate synthase activity (marker of mitochondrial abundance; n=8 per group). (F) Isolated cerebral mitochondrial ATP production rate (MAPR; n=9 per group) and cytochrome c oxidase (COX; complex IV) activity (marker of mitochondrial oxidative capacity n=8 per group). (**G**) Isolated cerebral mitochondrial reactive oxygen species (H_2_O_2_) production rate (n=9 per group) and superoxide dismutase 2 (SOD2) activity (marker of mitochondrial antioxidant defense; n=8 per group). (**H**) Working memory measured by 7-minutes spontaneous alternation behavior testing was quantified as % of maximum possible correct alternations (n=14 per group). (**I**) Mice were familiarized to a Y-maze for 5 minutes with one arm closed off, and then short-term memory was measured 3 hours later by assessing the time spent exploring the novel arm (now opened) during a 5-minute test (n=14 per group).

To determine whether insulin signaling in the brain causally regulates mitochondrial function and cognition, we generated conditional neuron-specific insulin receptor knockout (NIRKO) mice by administering tamoxifen to InsR^flox/flox^ mice expressing Nestin-CreERT2 (Figure S3A). Four weeks after tamoxifen induction, NIRKO mice exhibited significantly reduced InsR mRNA expression and InsR protein in the whole brain compared to Cre- littermate controls (Figure 4A), with no off-target reduction in InsR protein in peripheral tissues (Figures 4B–C). Systemic glucose metabolism was unaffected by neuron-specific InsR deletion, as NIRKO and Cre- mice displayed comparable responses during both glucose and insulin tolerance tests (Figure 4D), and no differences were observed in body composition, food intake, energy expenditure, or ambulatory activity (Figure S3B), consistent with findings from prior non-conditional transgenic models targeting neuronal insulin receptor loss under standard diet^64, 65^. Despite normal systemic metabolic parameters, NIRKO mice exhibited marked mitochondrial impairments in the brain. Cerebral mitochondrial DNA (mtDNA) copy number (Nd1 and Nd4) and citrate synthase (CS) activity were significantly reduced in NIRKO mice, indicating diminished mitochondrial content (Figure 4E), consistent with previous observations in diet-induced and genetic models of brain IR^64, 65^ and aging humans^66^. These results were consistent across all isolated hippocampal and cortical regions (Figure S3C). High-resolution respirometry of cerebral mitochondria revealed reduced ATP production rate (MAPR) concomitant with lower cytochrome c oxidase (COX) activity in NIRKO mice (Figure 4F). Moreover, COX activity was also decreased in the hippocampal and cortical mitochondria of NIRKO mice (Figure S3C), reflecting impaired oxidative phosphorylation capacity. Cerebral mitochondrial reactive oxygen species (H_2_O_2_) production was elevated (Figure 4G), while mitochondrial antioxidant capacity, as measured by superoxide dismutase 2 (SOD2) activity in cerebrum, hippocampus, and cortex, was reduced (Figures 4G and S3C), indicating increased oxidative stress, a well-documented consequence of disrupted brain insulin action^67^.

To assess cognitive consequences of neuron insulin receptor deletion, we tested NIRKO mice in two widely validated hippocampal-dependent tasks^68^. First, spontaneous alternation behavior in a Y-maze was used to assess working memory, defined as the capacity to retain and update spatial information during ongoing exploration. NIRKO mice exhibited a significantly lower percentage of correct alternations than Cre- controls (Figure 4H), indicating reduced working memory performance. Second, we evaluated short-term spatial recognition memory using a Y-maze novel arm test, in which mice were reintroduced to the maze three hours after initial exposure. Cre- mice preferentially explored the newly opened arm, whereas NIRKO mice failed to distinguish the novel arm (Figure 4I), suggesting deficits in short-term memory encoding or retrieval. These behavioral impairments parallel the cognitive deficits commonly observed in individuals with T2DM, who often show reduced performance in tasks requiring working memory, executive control, and spatial navigation^69^. These cognitive domains depend on coordinated activity across the frontal cortex and medial temporal lobe structures which in humans is specialized for scene processing and spatial context representation^70^. Supporting this translational link, resting-state fMRI in our human cohort revealed that IR was associated with reduced functional connectivity between frontal region and limbic brain structures implicated in these cognitive processes, particularly those involving the posterior dorsal cingulate and middle frontal cortices (Figure 2F). Thus, mitochondrial and cognitive impairments in NIRKO mice provide mechanistic support for disrupted large-scale brain network dynamics observed in humans with IR.

### 40 Weeks of Metformin Enhances Brain Glucose Uptake and Functional while Preventing Age-related Brain Atrophy.

**Figure 5:**
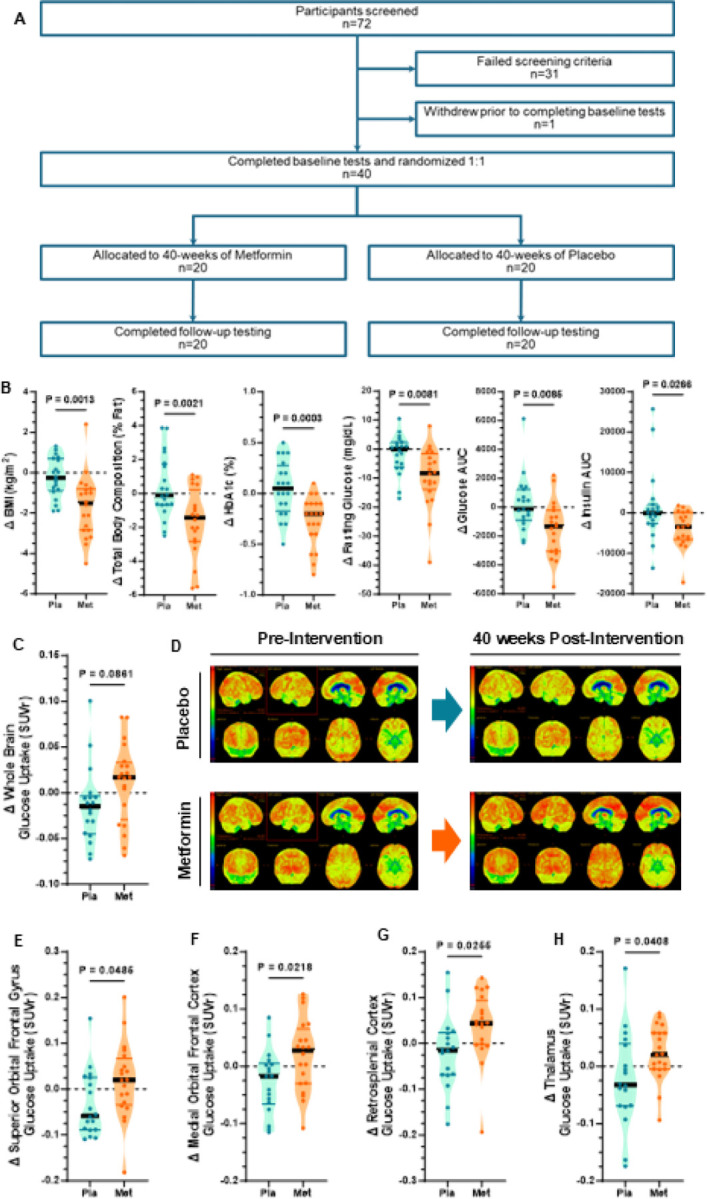
40 Weeks of Metformin Improves Glucose Uptake in Specific Brain Regions. (**A**) After passing inclusion and exclusion criteria, 40 participants with IR completed baseline testing (Cohort 2 from Supplemental Figure 1A) and were randomized in a double-blind fashion to 40-weeks of either 2000mg/day metformin or placebo. Baseline tests were repeated in all participants after 40-weeks of intervention. (**B**) The change (Δ) in in body mass index (BMI, n=20 per group), body composition (% fat, n=20 per group), HbA1c (n=20 per group), controlled fasting glucose (n=20 per group), and glucose and insulin area under the curve (AUC during oral mixed meal tolerance test, n=19 per group) after 40-weeks of either Placebo (Pla) or Metformin (Met) is shown as median (Black line) with participants represented as individual dots. (**C**) A statistical trend (P=0.086) towards group differences in the intervention-effect on whole brain glucose uptake, displayed as relative standardized uptake value (SUVr). (**D**) Standardized 3-dimensional FDG SSP (Stereotactic Surface Projection) intensity maps demonstrate Pre- and Post-Intervention (Placebo vs Metformin) glucose uptake across the whole brain. (**E-H**) Brain regions with statistically different intervention-induced change (Δ) in glucose uptake. Sample sizes were n=18 for Placebo and n=20 for Metformin for ^18^FDG-PET analysis. Two-way ANOVAs were used to compare group differences in Pre vs Post Intervention values and the p-value of the interaction effect is displayed.

We next aimed to determine if enhancing insulin sensitivity could prevent or reverse the deleterious impacts in the brain of people with IR. Metformin is a widely prescribed, low-cost generic medication, making it more accessible than newer treatments such as GLP1 receptor agonists or SGLT2 inhibitors^71^, and carries a lower risk of hypoglycemia than agents like insulin or sulfonylureas^72, 73^. Thus, we investigated whether metformin could prevent or mitigate dementia in the growing population of older adults with IR. Forty participants (Cohort 2, Figure S1B) with IR were randomized 1:1 in a double-blind fashion to either 40-weeks of 2000 mg/day (or as low as 1500 mg/day based on tolerance) metformin or placebo (Figure 5A). Baseline characteristics were similar between groups in body composition, blood pressure, and glucose homeostasis (Table 1), aside from higher triglycerides in the metformin group (P = 0.013) and slightly higher AST in the placebo group (P = 0.049). Overall, at baseline the groups shared a similar metabolic profile of early stages of IR.

After participants were randomized to treatment groups, monthly check-ups were used to assess adverse events (Figure S4A) as well as plasma metformin levels to confirm compliance in the metformin group (Figure S4B). Plasma metformin was also measured at baseline and at 40-weeks in the placebo group, and no participant in the placebo group had detectable levels of plasma metformin. Since metformin is known to lower plasma vitamin B12 levels^32^, potentially impacting cognitive function^33^, all participants were prescribed vitamin B12. Plasma vitamin B12 concentrations were measured at baseline, and again at 1, 3, and 6 months, as well as at the end of the study (Figure S4C), to ensure that B12 levels were above the threshold for clinical deficiency (defined as 180 ng/L). As expected, 40-weeks of metformin, but not placebo, reduced BMI, body fat%, fasting glucose, insulin, c-peptide, HOMA-IR, and HbA1c (Figure 5B). The metformin group, but not the placebo group, displayed improvements in glucose, insulin and c-peptide area-under-the-curve (AUC) following an oral mixed meal tolerance test (Figure 5B and S4D-G). From these values, the oral minimum model^74^ was used to calculate insulin sensitivity (SI) and shows a trend (P=0.057) towards a significant improvement after 40-weeks in the metformin group but not the placebo group (Figure S4H). Pancreatic beta cell function, based on the oral minimum model, was not impacted by 40-weeks of either metformin or placebo (Figure S4H). Thus, these data demonstrate that in our study cohort the expected improvement in whole body insulin sensitivity was observed with long-term metformin, but not placebo.

Next, we aimed to assess if there were brain-related changes that occur alongside the long-term metformin-induced increase in insulin sensitivity. We found a trend (P=0.086) towards improved whole brain glucose uptake with metformin, but not placebo (Figures 5C–D). Of interest, brain regions low in insulin receptors such as the mid-brain are not expected to increase glucose uptake with insulin sensitizer therapy^75^ and therefore the lack of generalized increase in glucose uptake is not unexpected. There was a significantly (P<0.05) greater 40-week change in glucose uptake with metformin than placebo in two prefrontal regions (superior orbital frontal gyrus, medial orbital frontal cortex), as well as the retrosplenial cortex and thalamus (Figures 5E–H) and all these regions are known to be rich in insulin receptors. The 40-week change in glucose uptake in other brain regions, though not statistically different, was almost always trending greater in the metformin group than the placebo group (Figure S5A), suggesting metformin may enhance fuel availability throughout much of the brain in individuals with IR.

**Figure 6:**
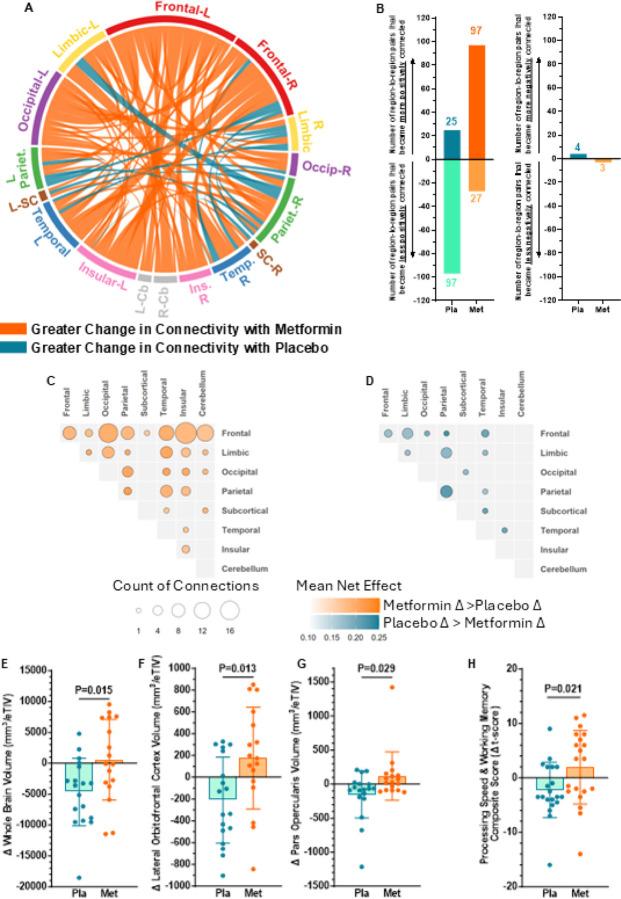
40 weeks of metformin enhances functional connectivity and prevents age-related brain atrophy. (**A**) Chord diagram represents all significant (FDR<0.05) between-group differences (Metformin vs Placebo) in the 40-week change in resting-state functional connectivity measured by fMRI (n=20 per group), arranged by hemisphere and lobe. Greater change in functional connection in the metformin group (100 connections) and placebo group (25 connections) is shown as orange and teal ribbons, respectively. Ribbon width corresponds to the magnitude of group differences in functional connectivity, computed as the Fisher z-transformed correlation difference (Δz) and weighted by statistical significance (αΔz). Occip. = Occipital, Pariet. = Parietal, Temp. = Temporal, SC=Subcortical, Ins. = Insula, and Cb = Cerebellum. (**B**) Number of region-to-region connections (based on fMRI) that became more positively (bars going up, left panel) or less positively (bars going down, left panel) connected or more negatively (bars going up, right panel) or less negatively (bars going down, right panel) connected with either placebo (teal) or metformin (orange). (**C**) The functional connections that became more positive with metformin were included in bubble diagram demonstrating the frontal lobe was largely responsible for significant group differences altered lobe-to-lobe connectivity. (**D**) The functional connections that became more positive with placebo were included in bubble diagram demonstrating fewer positive connectivity differences observed in response to placebo than metformin. (**E**) The change (Δ) in whole brain (excluding ventricles) volume (normalized to estimated intracranial volume, eTIV) after 40-weeks of either Placebo (Pla) or Metformin (Met) is shown as means (bars) with participants represented as individual dots. (**F-G**) The change (Δ) in the white matter volumes of the Lateral Orbital Frontal Cortex and the Pars Opercularis after 40-weeks of intervention. Sample sizes were n=18 for Placebo and n=17 for Metformin for volumetric analysis. (**H**) The change in composite measures of Processing Speed and Working Memory subtests from the Patient-Reported Outcomes Measurement Information System (PROMIS) instruments in the NIH Toolbox-Cognition. The change in the T scores from baseline to 40-weeks was compared between placebo or metformin (n=20 per group). Data are presented as mean ± SD. Dots represent individual participants.

Metformin also led to more pronounced alterations in functional brain connectivity than placebo (Figure 6A). Specifically, the increases in connectivity observed with metformin were largely driven by strengthened positive functional connections (Figure 6B), reflecting greater synchrony between brain regions. The most substantial metformin-induced changes in connectivity were observed between regions in the frontal lobe and other cortical areas (Figure 6C). Functional network analysis revealed that the regions involved in executive control network were primarily responsible for metformin-induced changes in functional connectivity (Figure S5B). In contrast, changes in connectivity with 40 weeks of placebo, which more reflects age-related changes rather than treatment effects, were far less pronounced (Figures 6D and S5C) versus the metformin group. These findings suggest that metformin helps to preserve or enhance brain network integrity in regions vulnerable to age-related decline.

Strikingly, although the placebo group experienced significant reduction in whole brain volume, which is consistent with age-related brain atrophy in people with IR^35^, metformin completely prevented reductions in brain volume over the 40-week period (Figure 6E). The prevention of brain atrophy with metformin was also statistically significant in two key prefrontal regions, the lateral orbitofrontal cortex and the par opercularis (Figures 6F–G), but other regional brain volumes were unchanged with 40 weeks of metformin vs placebo (Figure S5D). Together, these data suggest that pharmacologically maintaining adequate supply of glucose to specific brain regions can enhance functional connectivity and prevent IR-mediated brain atrophy during aging.

To determine whether the metabolic and structural brain benefits of metformin translate into cognitive benefits, we also assessed cognitive function. The 40-week change in a composite score of processing speed and working memory was significantly greater with metformin, than with placebo (Figure 6H). There were no significant improvements in the metformin group on other cognitive outcomes (composites or individual subtests). As the current study was primarily focused on the impact of metformin on brain biology, it was not sufficiently powered to detect changes in all cognitive outcomes (Figure S6). These findings indicate that the enhancements in regional glucose uptake, strengthened positive functional connectivity—particularly within the frontal lobe and executive-control networks—and preservation of prefrontal volume with metformin may each converge to enhance cognition in older adults with IR. Processing speed and working memory are among the earliest cognitive domains to decline with IR and aging^76^, and they critically depend on efficient frontal lobe network function and adequate cerebral energy supply^77^. Our data therefore support a model in which metformin, by improving insulin sensitivity and maintaining fuel availability, restores network efficiency and attenuates age-related slowing of cognitive processing.

## DISCUSSION

The current study demonstrates that IR diminishes brain glucose uptake in prefrontal, temporal, and parietal regions that are critical for important cognitive function. We also observed lower global brain volume with regional atrophy, most notably in the hippocampus, important for forming new memories and long-term storage of memory, and white-matter tracts that mediate inter-regional communication. Functionally, IR was linked to weakened connectivity among networks essential for higher-order cognition relative to insulin sensitive individuals. Complementary experiments in conditional NIRKO mice revealed reduced brain mitochondrial ATP production and increased reactive oxygen species, accompanied by deficits in hippocampal- and prefrontal-dependent tasks, providing mechanistic context for the human findings. To test modifiability of IR-induced brain deficits, a 40-week metformin intervention versus placebo in older adults with IR improved whole-body insulin sensitivity, increased brain glucose uptake in areas intimately involved in cognition, and enhanced structural integrity and functional connectivity in cognitive circuits. Metformin attenuated IR-related atrophy and selectively increased cortical volumes (e.g., lateral orbitofrontal cortex, pars opercularis). Despite the relatively short intervention, participants exhibited small but measurable gains in processing speed and working memory with metformin. Together, these data suggest that improving insulin sensitivity through pharmacologic means may represent a promising strategy to preserve or restore brain function in individuals with IR.

We consider brain glucose uptake, as measured by ^18^F-FDG PET, to reflect mitochondrial energy metabolism, given that glucose is the brain’s primary and obligatory fuel under normal physiological conditions. Insulin is a critical regulator of skeletal muscle mitochondrial biogenesis and function in humans^78, 79^ and IR and deficiency render mitochondrial oxidative phosphorylation inefficient despite adequate glucose availability, resulting in decreased ATP production and increased ROS emission^78, 79^. We have observed similar deficits in brain IR in preclinical models, which can be improved by insulin-sensitizing interventions like aerobic exercise and metformin^22^. Further, our NIRKO mouse model confirmed that loss of neuronal insulin signaling impairs mitochondrial function across brain regions, with reductions in ATP production and elevated ROS. Moreover, insulin receptor antagonists blocked insulin-induced mitochondrial enhancement^22^, supporting the idea that insulin plays a key role in maintaining brain oxidative phosphorylation.

The current findings show that IR is associated with reduced functional connectivity between brain regions involved in cognition particularly within and between limbic regions of the frontal and temporal lobes, important for executive control networks, which are particularly vulnerable to aging and the development of AD^56^. The posterior cingulate cortex emerged as a key hub within these networks, demonstrating many strong positive functional connections in insulin-sensitive individuals, with markedly diminished connectivity observed in those with IR. This region exhibited strong and widespread connections to multiple frontal, temporal, and parietal areas, reflecting its integrative role across brain systems. These patterns align with prior studies linking prediabetes and T2DM to reduced network integration^55, 80^. The current study demonstrated that 40 weeks of metformin, which significantly improved insulin sensitivity and enhanced brain glucose uptake, strengthened functional connectivity, especially among frontal regions involved in executive control and emotional regulation. In contrast, the placebo group exhibited declines in connectivity, suggesting deterioration in network integrity without insulin sensitization, with 99 connections becoming significantly weaker and only 26 becoming stronger. In comparison, metformin-treated participants showed 98 connections becoming stronger and only 28 weaker, indicating that insulin sensitization can restore large-scale network communication, particularly in memory-related systems.

Importantly, we found that metformin enhanced brain glucose uptake, particularly in prefrontal areas enriched with insulin receptors and involved in executive function, learning, and memory^81^. This suggests that increased cerebral energy metabolism may be a key mechanism underlying the observed improvements in brain structure and function. We hypothesize that these connectivity enhancements are driven by increased cerebral glucose uptake and, consequently, greater ATP availability, which supports synaptic transmission and network integration^82^. Our findings in NIRKO mice reinforce this interpretation, showing that loss of neuronal insulin signaling impairs mitochondrial oxidative phosphorylation, reduces ATP production, and elevates ROS emission, which may have contributed to reduced working memory and spatial reference memory. Additionally, preclinical models of IR demonstrate that metformin restores mitochondrial function, increasing ATP production while reducing oxidative stress^22^. Taken together, these findings add to the growing body of evidence that the brain is a highly insulin-sensitive organ, especially in regions critical for cognition, and that insulin signaling plays a central role in regulating mitochondrial metabolism, neuronal connectivity, and structural integrity^83^.

Of interest, other measures of weight loss such as bariatric surgery^84, 85^, GLP-1 receptor antagonists^86^, GLP-1/GIP dual agonists^87^ and SGL 2 inhibitors^88^ have been reported to reduce dementia in obese people with IR and/or T2DM. A common factor that seems to contribute to the cognitive effect in all the above studies is weight loss, and its impact on energy balance causing an improvement in insulin sensitivity. Although these drugs are increasingly prescribed with impressive effects on glycemic control and other cardio-metabolic benefits, these drugs and bariatric surgery are associated with substantially higher weight loss than metformin.

Brain IR has been identified as a risk factor for cerebral atrophy^36^. We found that IR was associated with generalized reductions in brain volume, with particularly pronounced atrophy in white matter regions critical for interregional communication and information processing. Remarkably, 40 weeks of metformin treatment led to structural improvements in the brain. Compared to the placebo group, participants receiving metformin showed significant increases in total brain volume, including targeted increases in the lateral orbitofrontal cortex, an area involved in decision-making, learning, and reward integration^53^, as well as in the pars opercularis, which forms the posterior portion of Broca’s area and plays a central role in language production^89^. These structural improvements coincided with increased glucose uptake in multiple subdivisions of the orbitofrontal cortex, which likely represents enhancement of mitochondrial metabolism due to metformin^10, 78^. Preclinical studies support our interpretation of the impact of insulin sensitization by metformin, on improved ATP production in brain areas rich in insulin receptors^22^. This improved mitochondrial function is critical for maintaining proteome homeostasis and neuronal integrity^90^. Conversely, IR and insulin deficiency have been shown to impair oxidative phosphorylation, promoting oxidative stress and protein damage in the brain^79^. Our findings demonstrate that the structural atrophy observed in insulin-resistant individuals may be prevented or reversed by improving insulin sensitivity and suggests that improved mitochondrial efficiency and cellular energy availability may underlie the neuroprotective effects of insulin sensitization.

IR has been linked to cognitive decline in older adults, with elevated IR increasing the risk for dementia^91, 92, 93^. In our preclinical model, experimentally induced IR led to deficits in working memory and spatial recognition, underscoring a causal relationship between impaired insulin signaling and cognitive dysfunction. Extending these findings to humans, participants receiving metformin showed significant improvements in measures of processing speed and working memory, compared to those receiving placebo. These measures reflect the capacity and efficiency of information processing, and their enhancement may support broader gains in executive function and memory performance in daily life despite not seeing specific effects on executive function and memory measures. Collectively, these findings support the hypothesis that improving insulin sensitivity may help preserve or restore cognitive function in individuals with IR.

### Limitations of Study

Several limitations should be acknowledged. First, the sample size was not sufficiently powered to detect changes across the full range of cognitive outcomes. Although metformin improved processing speed and working memory, larger studies will be necessary to assess its broader effects on cognition and quality of life measures. Additionally, the 40-week intervention period may have been too short to fully capture the long-term impact of metformin on brain structure and function. Second, our use of ^18^F-FDG PET to assess cerebral glucose uptake relies on the assumption that glucose uptake serves as a valid proxy for mitochondrial energy metabolism. While direct measurement of ATP production rate would be ideal, there are currently no sensitive and precise methods for assessing in vivo ATP synthesis rates in discrete brain regions, particularly those implicated in cognitive processing. While techniques such as magnetic resonance spectroscopy (MRS) with saturation transfer have been successfully applied to quantify maximal ATP production rates in skeletal muscle^94, 95^, and resting state liver (), these approaches have not yet been validated or optimized for use in the human brain. In prior studies, we measured cerebral energy metabolites to examine the effects of transient insulin deprivation^50^. However, due to the rapid turnover of ATP and phosphocreatine (PCr), these methods are not ideal for assessing long-term metabolic changes or comparing across individuals. Ideally, direct measurement of brain ATP production rates would offer definitive insights into cerebral energy metabolism. However, there are substantial technical challenges to quantifying ATP flux in specific brain regions. Since the first in-human study^96^, nearly all measurements of phosphorus metabolite fluxes (ATP, PCr, and inorganic phosphate [Pi]) in the human brain have been performed using direct surface coil localization. While convenient due to the increased signal obtained by coil, the inherent heterogeneous B1 field of surface coils lead to variable flip angles, and therefore variable metabolite saturation, within the adjacent brain parenchyma resulting in inaccurate measurements that differ based on the brain position beneath the coil. Chen *et al*.^97^ attempted to address this issue using the ISIS (image-selected in vivo spectroscopy) sequence to improve localization; however, the acquisition still relied on a surface coil, and the solution involved excluding signal from the periphery of the coil rather than eliminating the underlying variability. In principle, using a volume head coil, capable of producing a more homogeneous B1 field, would enable more accurate and reproducible flux measurements across brain regions. Unfortunately, such methods have not yet been sufficiently developed or validated for this purpose.

Despite the challenges, ^18^F-FDG PET remains a highly sensitive and spatially precise tool. Unlike skeletal muscle, heart, or liver, the human brain relies almost exclusively on glucose for ATP production under normal physiological conditions, except during prolonged fasting or insulin deficiency, when ketones may be temporarily utilized as an alternative substrate^75, 98^. Therefore, with the available established technologies and limitations in establishing innovative approaches to directly measure brain ATP production rate, the current study relied on glucose uptake as a valid surrogate for mitochondrial function in the brain, which is both physiologically appropriate and methodologically justified.

Lastly, although our study demonstrates parallel improvements in insulin sensitivity, brain glucose uptake, connectivity, and structure, we cannot definitively establish whether metformin’s effects on the brain were direct or secondary to systemic metabolic improvements. However, a growing body of preclinical evidence suggests that IR directly impairs mitochondrial function in the brain^22, 64^. Importantly, these mitochondrial deficits appear to be reversible through interventions that enhance insulin sensitivity, including aerobic exercise in humans with IR^99^ and metformin treatment in mouse models of diet-induced IR^22^. Together, these findings reinforce the hypothesis that improving insulin sensitivity, whether through pharmacological or behavioral strategies, has beneficial effects on brain metabolism and function.

## Conclusions

This study demonstrates that IR is associated with reduced glucose uptake, functional connectivity, and brain volume, whereas treatment with the insulin sensitizer metformin induced improvements in glucose uptake accompanied by structural preservation and improved neuronal connectivity. These findings are consistent with the hypothesis that impaired energy metabolism disrupts ATP-dependent functional connectivity between brain regions critical for cognition in insulin-resistant individuals. Importantly, IR in mice and humans, as well as metformin in humans, consistently affected the frontal cortex, particularly prefrontal regions that anchor executive control and working memory. Additionally, we found that IR was associated with reduced total brain volume, including specific atrophy in cerebral white matter and the hippocampus, a structure essential for memory consolidation. Plasma glucose levels negatively correlated with whole brain, frontal white matter, frontal pole, and corpus callosum volumes, reinforcing the connection between metabolic dysfunction and structural brain deterioration. In contrast, 40 weeks of metformin treatment which improved in insulin sensitivity resulted in increased total white matter volume, including the lateral orbitofrontal cortex and pars opercularis, regions involved in decision-making, language production, and cognitive control. Although the study was not powered to detect changes across a broad range of cognitive outcomes, we observed improvements in processing speed and working memory in the metformin group compared to placebo. These findings suggest that even a relatively short intervention targeting insulin sensitivity may positively influence key cognitive domains.

In conclusion, IR in humans is associated with significant impairments in brain glucose metabolism, structural integrity, and network connectivity, particularly in regions that are critical for cognitive functions. Experimental animal models support the hypothesis that IR impairs brain mitochondrial oxidative phosphorylation, reducing ATP availability and increasing oxidative stress—changes that may underlie brain atrophy and domain-specific cognitive deficits. Metformin treatment reversed several of these deficits, highlighting the potential for insulin-sensitizing therapies to mitigate or prevent the adverse neurological effects of IR. These findings provide compelling evidence that targeting insulin sensitivity, through affordable, widely used treatments like metformin, may represent a viable strategy to prevent or reverse the debilitating neurological consequences of IR.

## STAR METHODS

KEY RESOURCES TABLE

### RESOURCE AVAILABILITY

#### Lead contact:

Further information and requests for resources and reagents should be directed to and will be fulfilled by the Co-Lead Contact, K. Sreekumaran Nair (nair@mayo.edu).

#### Materials availability:

This study did not generate any unique reagents.

#### Data and code availability:

Source data for the graphs are provided in Data S1. Supplementary Data File S1 represents an Excel file containing the values that were used to create all the graphs in the paper. Any additional information required to reanalyze the data reported in this paper is available from the lead contact upon request.

### EXPERIMENTAL MODEL AND STUDY PARTICIPANT DETAILS

#### Human experiments

##### Participants and study design:

The study design was approved by the Mayo Clinic Institutional Review Board and registered under Clinical Trials (Cohorts 1 and 2 under ClinicalTrials.gov Identifier NCT03733132; Cohort 3 under ClinicalTrials.gov Identifier NCT04158375). All participants were informed of the study procedures and provided written consent. A total of 74 participants, 39 women and 35 men, between 50–80 years of age across the four study cohorts underwent baseline testing. For each of the respective study cohorts, participants were selected based upon the following inclusion criteria: Cohort 1 - age 60–80 years, BMI 20–24.9 kg/m^2^, and fasting plasma glucose ≤ 95 mg/dL; Cohort 2 - age 60–80 years, BMI 25–38 kg/m^2^, and fasting plasma glucose 100–140 mg/dL; and Cohort 3 – age 50–75 years, BMI 28–38 kg/m^2^, and fasting plasma glucose 100–140 mg/dL. Exclusionary criteria for all cohorts included the following: use of hypoglycemic medications including metformin, active coronary artery disease or cardiovascular disease, abnormal renal function (defined as calculated GFR <60 mL/min/1.73 m^2^), abnormal liver function (defined by transaminase >2 times the normal upper limit), known active adrenal disorder, active gastroparesis, a lack of stability over the previous 2 months for certain medications (including antihypertensive, thyroid, anti-depressant, or lipid-lowering medication), uncontrolled thyroid disease (defined by undetectable TSH of > 10 mIU/L), abuse of alcohol or recreational drugs, arterial hypertension (resting diastolic blood pressure >90 mmHg and/or systolic blood pressure >160 mmHg) at the time of screening, oral steroid use, any metal in the body that could interfere with MRI, lack of English proficiency, use of beta blockers, and a Montreal Cognitive Assessment (MoCA) score at the screening visit < 22. Medical records were also screened for any documented diagnosis of cognitive impairment/dementia. Furthermore, participants were excluded from Cohorts 2 and 3 (Insulin Resistant cohorts) if they reported engagement in structured exercise ≥ 2 days per week. Conversely, participants in Cohort 1 (Healthy Controls) were recruited to participate if they reported participation in structured exercise training ≥ 3 days per week. Participants completed both inpatient and outpatient study visits. The inpatient visit included 3 days of standardized meals followed by an overnight visit in the Clinical Research Unit (CRU) at Mayo Clinic in Rochester, Minnesota, with a controlled fasted blood draw in the morning. Cognitive tests using the NIH Toolbox-Cognition were administered approximately 1 hr following the evening meal during the inpatient visit at the CRU. The outpatient visits included body composition assessment by dual-energy X-ray absorptiometry (DEXA), ^18^FDG-PET scans of the brain, brain MRI, brain fMRI.

Following baseline testing, participants in Cohort 2, which included a total of 40 participants (20 women and 20 men) with IR (based on screening glucose ≥ 100mg/dL), were randomly assigned under double-blind conditions to receive either a 2000 mg/day regimen of metformin or an equivalent placebo dose for 40 weeks. Participants returned for monthly check-in appointments to assess body weight, refill study prescription, and obtain a blood draw to assess plasma vitamin B12 and metformin levels. After the 40-week intervention, participants repeated all baseline testing. The baseline and follow-up inpatient visit for this cohort included an oral mixed meal tolerance test following the controlled overnight fast.

#### Mouse experiments

##### Mouse Brain Insulin Receptor Knockout Model:

Mice with the insulin receptor (InsR) gene flanked by loxP sites (InsRf/f) (generated by Ronald Kahn, Joslin Diabetes Center) were crossed with the NestinCreERT2 mouse line (Stock #016261; C57BL/6-Tg(Nes-Cre/ERT2)KEisc/J; Jackson Laboratory) to generate brain-specific insulin receptor knockout (NIRKO) mice. Mice negative for Cre but either heterozygous or homozygous for the floxed InsR allele were used ad Cre-negative controls. Mice were housed with ad libitum access to food (13% energy as fat, PicoLab Rodent Diet, 5053) and water and kept on a 12-h (8 a.m. to 8 p.m.) light-dark cycle in a humidity- and temperature-controlled room. Genomic DNA was isolated from a tail snip, the Cre and InsR genes were amplified using the appropriate primer sequences (Cre: Fwd 5’- GCGGTCTGGCAGTAAAAACTATC – 3’, Rev 5’ – GTGAAACAGCATTGCTGTCACTT – 3’; InsR: Fwd 5’ - GATGTGCACCCCATGTCTG - 3’, Rev 5’ - CTGAATAGCTAGACCACAG - 3’), and PCR products were resolved on a 1.5–2% agarose gel and visualized under UV light to asses band patterns corresponding to each allele. To induce recombination and knock out of insulin receptor expression in the brain, tamoxifen (Sigma-Aldrich) was dissolved in corn oil at a concentration of 20 mg/mL and administered via intraperitoneal (IP) injection at a dose of 75 mg/kg body weight. Injections were performed once daily for five consecutive days starting at 12 weeks of age. Cre-negative mice received identical tamoxifen injections to control for off-target effects of the drug. Following tamoxifen induction, mice were returned to standard housing conditions and monitored regularly. All animals were studied four weeks following tamoxifen injections (i.e., at 16 weeks of age) to allow sufficient time for recombination and subsequent reduction of insulin receptor expression in the brain. This timeline was selected based on prior studies demonstrating efficient recombination and functional knockout using the Nestin-CreERT2 system^100^. Mice were euthanized via pentobarbital overdose. Mice were fasted for 6 hours prior to sacrifice. Depending on the analysis, the cerebrum, hippocampus, or cortex were excised per a mouse brain atlas^101^.

### METHOD DETAILS

#### Human experiments

##### Screening Visit:

At the screening visit participants met with a study team member who described all procedures, then participants provided written consent. A fasting blood sample was obtained for plasma glucose and comprehensive chemistry panel (including CBC, serum creatinine, liver function tests and TSH, and lipid panel), a general medical examination was performed (including vital signs), and the MoCA test was administered. Hemoglobin A1c was obtained if not available in the participants medical record in the 3 months prior to the initial study visit.

##### Outpatient Study Visits:

###### Body Composition Testing (Outpatient Study Visit 1):

Within 60 days following the initial screening visit, eligible participants returned to the Clinical Research Unit (CRU) at Mayo Clinic for body composition analysis (DEXA) and to meet with a registered dietician to discuss food preferences for weighed-meals (based on Harris-Benedict equation).

###### Brain Scans (Outpatient Study Visit 2):

Approximately 7 days after body composition testing, participants underwent MRI, fMRI, and ^18^FGD-PET scans of the brain in the morning following an overnight fast.

MRI Scans were performed in the MRI unit at the Mayo Clinic on a 3 Tesla Siemens Skyra equipped with a 32-channel head coil and the multi-nuclear option (MNO) running VE11C software. To measure structural information associated with regional brain volume a sagittal 3D MPRAGE sequence with 0.7mm isotropic voxels (TR=2400, TE=2.57, TI=1100, FA=8) was used to acquire high-resolution structural data that would also be utilized for intracerebral functional connectivity. Moreover, an axial 2D symmetric multi-slice (SMS) diffusion tensor imaging (DTI) sequence with 60 diffusion directions, 5 B0 acquisitions and 2mm isotropic voxels (TR=3000, TE=73, FA=90, ETL 43, both A-P and P-A phase encoding for B0 images) was used to acquire white matter integrity data. MPRAGE was analyzed by converting to Neuroimaging Informatics Technology Initiative volume using mri_convert, then processed using recon_all with manual inspection of data. FC matrix calculations were tabulated after obtaining cortical and subcortical parcellations via Freesurfer N27 template.

To assess functional connectivity (FC) imaging was conducted using an axial 2D echoplanar imaging sequences with isotropic voxels at 3 mm (FA, 90 degrees; TE, 30 ms; TR, 3000ms; no. slices, 52; 116 volumes; volumes; 5 minutes and 48 seconds). The participants were asked to lie still while undergoing the assessment. In addition, global brain blood flow measurements including axial 3D-pseudo continuous arterial spin labelling (pCASL) sequence (WIP 818) with 4 mm isotropic voxels (TI1=1800, TI2=3600, iPat ≤ 2) was used to acquire quantitative blood flow measurements (flow in ml/100g/min) throughout the brain. A post-label delay of 1800 ms was used. fMRI images were preprocessed using CONN functional connectivity toolbox. This algorithm specifically, discarded the first 10 vol for steady-state magnetization, slice time correction, realignment, outlier detection, segmentation, and direct normalization to MNI template space, smoothing with a gaussian kernel of 6 mm full width, half maximum, nuisance regression for white matter, and denoised for 6 head motion parameters with their first and second-order derivatives, and a bandpass filtered in the 0.01–0.1 Hz frequency to reduce low–frequency drift and noise effects^102^. Following this process, the images were parcellated into cortical and subcortical areas using Harvard-Oxford atlas, CONN’s network parcellations atlas which was generated using independent component analysis (ICA) on the HCP dataset (N = 497) and Mayo Clinic intrinsic connectivity networks identified in a sample of healthy older participants from MCSA. The mean blood-oxygen-level, dependent (BOLD) time series within each region of interest (ROI) of the atlas were extracted^103^.

Cerebral glucose uptake was measured by ^18^fluorodeoxyglucose positron emission tomography (^18^FDG-PET). The PET scans were acquired utilizing a LYSO PET/CT scanner (DRX, GE Healthcare, WI). After injecting the participants with approximately 8 mCi (range 7–10 mCi) of FDG followed by a 30-min period to allow for uptake, PET was acquired for 8 minutes which consisted of four 2-minute frames after a low-dose CT image. The PET scans underwent routine corrections, and reconstructed sinograms were formatted into a 256 mm field of view (FOV) with pixels sized at 1.0 mm and slices measuring 3.3 mm in thickness. Attenuation correction utilized a helical CT image captured prior to FDG administration, accompanied by model-based scatter correction. After preprocessing, images underwent a voxel-wise, single-subject statistical analysis with a comparison to healthy sex- and age-matched controls provided by the CortexID software^104^ with an output of individual patients’ VOI z-scores and z-score maps presented as 3-dimensional Stereotactic Surface Projection (3D-SSP) images of the cortex. The Cortex ID normal database contains more than 100 subjects with no memory or other cognitive complaints and no evidence of neurodegenerative diseases aged 42–89. These statistical maps were subsequently analyzed to detect metabolic alterations between groups and pre- vs post-intervention.

An additional in-house fully automated image processing pipeline was used for FDG PET quantitative image analysis. Each participant’s FDG PET was registered to their MRI using built-in function in AFNI software^105, 106^ to co-register PET to MRI and unified segmentation on the MRI to produce spatial normalization parameters and a labeled atlas in native subject MRI space for each participant. FDG PET standardized uptake value ratios (SUVr) were then calculated for whole brain cortical ROIs defined on MRI using an in-house version (“MCALT ADIR122”) of the automated anatomic labelling atlas from both hemispheres normalized to pons uptake as previously described^107^.

##### Inpatient Study Visit:

###### Controlled Fasting Blood Draw:

Since the fasting blood draw that was collected to screen for glucose tolerance (healthy control or insulin resistant) was based on the participant’s self-reported last dietary intake (length of overnight fast) and level of physical activity (or inactivity), a more tightly controlled fasting blood collection was obtained on a separate inpatient study visit. Prior to this study visit, participants were provided with 3 days of standardized weighted meals (20% protein, 30% fat, 50% carbohydrate) based on the Harris-Benedict equation to maintain body mass. On the third day of weighted meals, participants reported to the CRU at 1700h, were provided a final weight maintenance meal at 1800h, and fasted overnight for 14hr before a blood draw was obtained to assess fasting glucose, insulin, and c-peptide. Participants remained inactive during this 14hr fast.

###### Cognitive Assessment:

At 1900h (1 hour after the final weight maintenance meal), the computerized NIH Toolbox-Cognition (V2) was used to measure cognitive outcomes. All participants were fluent/native English speakers and all tests were administered in English. The NIH Toolbox-Cognition Battery is composed of 7 core tests (~30 minutes) including the Dimensional Change Card Sort (DCCS) Test (executive function), the Flanker Inhibitory Control Test (executive function), the Picture Sequence Memory Test (episodic memory), the Picture Vocabulary Test (vocabulary), the Oral Reading Recognition Test (reading), the List Sorting Working Memory Test (working memory) and the Pattern Comparison Processing Speed Test (processing speed). Fully-corrected T-scores (mean = 50, SD = 10) that adjust for age, sex, education and race/ethnicity were used for all measures. In addition to examining individual task scores, three composite scores provided by the NIH Toolbox (Fluid Cognition Composite, the Crystallized Cognition Composite, and the Total Cognition Composite) were examined. The Fluid Cognition Composite reflects executive function, processing speed, working memory, and episodic memory, and includes scores from the DCCS, Flanker, Pattern Comparison Processing Speed, List Sorting Working Memory, and Picture Sequence Memory Tests. The Crystallized Cognition Composite captures acquired knowledge and language skills and includes scores from the Picture Vocabulary and Oral Reading Recognition Tests. The Total Cognition Composite represents overall cognitive functioning and represents performance across all 7 core tests. A fourth Processing Speed/Working Memory Composite score was generated post-hoc by taking the average performance across the Pattern Comparison Processing Speed and List Sorting Working Memory tests.

###### Oral Mixed Meal Tolerance Test:

At 0800 following the overnight fast, participants in cohort 2 consumed a standardized mixed meal shake composed of 20% protein, 50% carbohydrate, and 30% fat, and comprising 25kcal per kg of the individual’s resting total lean mass (measured by DEXA). Arterialized blood was collected before meal ingestion (T0) and at intervals over the 4 h following meal ingestion. At each timepoint, glucose was measured in freshly isolated plasma samples using a Yellow Springs Instrument (YSI) analyzer (YSI Inc, Yellow Springs, OH). EDTA plasma from each timepoint was stored at −80°C for the measurement of insulin using a one-step immunoenzymatic Access Ultrasensitive Insulin assay by the Mayo Clinic Immunochemical Core Laboratory (ICL). Serum samples from each time point were stored at −80°C for subsequent measurement of C-peptide using the Roche 2-site immunometric assay with electrochemiluminescent detection by the ICL.

Insulin sensitivity (SI) and β-cell responsivity (Φ) were estimated using the oral glucose and C-peptide minimal models, respectively^74, 108, 109, 110^. The disposition index (DI) was calculated as the product of SI and Φ^74^. Individuals for which the minimal models could not be resolved were excluded from the analysis.

##### Functional Connectivity Data Processing

Brain connectivity comparisons were derived from FDR-corrected statistically significant connections only. Seed and cluster regions were mapped to FreeSurfer anatomical labels using the 2009 atlas. Brain regions were then categorized into: 1) hemispheres: Left and Right; 2) eight anatomical divisions: Frontal, Temporal, Parietal, Occipital, Limbic, Cerebellum, Subcortical, and Insular cortices; and 3) nine functional networks: Auditory, Cerebellar, Default Mode, Dorsal Attention, Executive Control, Salience, Sensorimotor, Subcortical, and Visual. While anatomical divisions and network designations vary across studies, our classification scheme follows established approaches used in peer-reviewed connectivity analyses^111, 112, 113^.

Functional connectivity in fMRI reflects the temporal correlation of blood oxygen level-dependent (BOLD) signal fluctuations between brain regions, indicating coordinated neural activity. Positive connectivity (positive Fisher z-transformed correlation coefficients) indicates temporally correlated BOLD signals suggesting coordinated activity, while negative connectivity (negative Fisher z-transformed correlation coefficients) represents inversely correlated signals potentially indicating competitive or inhibitory relationships. Stronger connectivity reflects higher correlation coefficients between regional BOLD time series, suggesting more consistent coordination, while weaker connectivity indicates less consistent coordination. Both positive and negative connectivity, whether strong or weak, are required for healthy brain function – their functional significance depends on the specific regions and cognitive contexts involved.

A threshold of ±0.05 was applied to Fisher z-transformed correlation coefficients to distinguish meaningful positive and negative connectivity. Values within this threshold (between −0.05 and +0.05) were considered effectively zero, as correlations of this magnitude represent negligible functional relationships regardless of sign. This approach ensures that when comparing between subjects or across time within subjects, meaningful changes in functional coupling patterns from weak to strong connectivity (or vice versa) are identified rather than misinterpreting noise-level fluctuations as transitions from positive to negative connectivity (or vice versa). Raw connectivity values were used for all calculations; thresholding was applied only for categorical interpretations.

Cross-section connectivity differences were calculated as between-group mean differences at baseline: IR - HC, where positive values indicated stronger connectivity in insulin-resistant participants and negative values indicated stronger connectivity in healthy controls. Longitudinal treatment effects were quantified by subtracting the treatment effect of placebo (Placebo post – Placebo pre) from the treatment effect of metformin (Metformin post – Metformin pre). This approach highlights treatment-specific changes while controlling for time-related effects and random differences in baseline values.

Positive values indicate greater connectivity change with Metformin treatment and negative values indicate greater connectivity change in the Placebo group.

Data were processed and analyzed using R (version 2024.12.1) and tidyverse, chord diagrams were crezated using circlize^114^, and ggplot2 was used for bivariate heat maps. The network diagram of specific connected pairs in the frontal, limbic, and temporal regions in the baseline comparison were created using Cytoscape (version 3.10.3). All other connectivity figures were created in GraphPad Prism 10.

##### Statistical Analysis:

The primary aims of this human study were to investigate: 1) how brain function and physiology is influenced by whole body metabolism in older people with and without IR, and 2) if 40 weeks of metformin treatment (in older people with IR) enhances brain function and physiology. To accomplish the first aim, we measured regional brain glucose uptake, volumes, and functional connections, as well as controlled fasting blood markers of insulin sensitivity in 74 participants (56 insulin resistant, IR; and 18 insulin sensitive healthy controls, HC). These brain measures were compared between groups (HC vs IR) and correlated with insulin sensitivity markers across all participants. A subset of 40 participants in the IR group were also enrolled in a double-blind randomized controlled trial and received 40-weeks of either placebo or metformin (n=20 per group). The effect of intervention (placebo or metformin) after 40-weeks (delta effect, Δ) on brain and whole body insulin sensitivity measured were compared between groups.

Unless otherwise stated, the results are expressed as means with individual data points shown to illustrate variability. Statistical analyses were performed using GraphPad Prism (version 8, GraphPad Software). To determine the significance of differences observed between HC and IR groups in various outcomes, we employed two-sided unpaired Student’s t-tests. Two-way ANOVAs were conducted to evaluate the significance of changes (Δ) in response to 40 weeks of placebo or metformin treatment. A significance level of p < 0.05 was considered statistically significant for all analyses. For fMRI analyses, the Benjamini-Hochberg procedure was applied to control the false discovery rate (FDR). The exact value of n within the figures is indicated in the figure legends.

For baseline correlations between brain outcomes (regional glucose uptake and volumes) and markers of insulin sensitivity, simple linear regression was performed. For both glucose uptake and volumetric analyses, identified regions were grouped based on their anatomical position (such as lobes for cortical regions and deep structures for subcortical areas), then the Benjamini-Hochberg procedure was applied to control the false discovery rate (FDR) within each anatomical area. Asterisks in heatmaps of linear regression analyses represent p-values that have passed FDR correction.

#### Mouse experiments

##### Oral Glucose Tolerance Test:

Oral glucose tolerance tests (OGTT) were conducted by orally administering glucose (2 mg/kg body weight) after a 12-hour fast. Blood samples for glucose measurements were obtained 0, 15, 30, 45, 60, 90, and 120 minutes after administration. Blood glucose levels were measured using an AlphaTrak 2 glucometer (Abbott Laboratories). Area under the curve (AUC) was calculated to evaluate glucose tolerance.

##### Insulin Tolerance Test:

Insulin tolerance tests (ITT) were conducted by IP insulin injection (0.75 mg/kg body weight) after a 2-hour fast. Plasma samples for insulin measurements were obtained 0, 15, 30, 45, 60, 90, and 120 minutes after administration. AUC was calculated to evaluate insulin tolerance.

##### Body Composition:

Body composition was measured using quantitative magnetic resonance imaging (EchoMRI-100; EchoMRI LLC, Houston, TX).

##### Comprehensive Laboratory Animal Monitoring:

Energy expenditure and metabolic phenotyping were assessed using a Comprehensive Laboratory Animal Monitoring System (CLAMS) equipped with an Oxymax Open Circuit Calorimeter System (Columbus Instruments, Columbus, OH, USA). Mice were individually housed in open-circuit indirect calorimetry chambers that continuously recorded oxygen consumption (VO_2_), carbon dioxide production (VCO_2_), respiratory exchange ratio (RER = VCO_2_/VO_2_), energy expenditure (kcal/hr), physical activity, and food intake. RER values were used to determine the resting metabolic expenditure (in kilocalories per kilogram per hour). Mice were acclimated to the chambers for 24 hours prior to data collection, and measurements were recorded every 15 minutes over a 48-hour period, including one full light/dark cycle. Environmental conditions were maintained at 22–24°C with 40–60% relative humidity and a 12:12-hour light-dark cycle, with ad libitum access to standard chow and water. Ambulatory, rearing, and total activity was measured and analyzed for light and dark periods by infrared beam breaks across the x-, y-, and z-axes. Ambulatory activity was defined as consecutive beam breaks in the x-axis, whereas total activity included all movements across axes. Food intake was continuously monitored by automated weight sensors integrated into the feeding trays.

##### Brain Mitochondrial Isolation:

Mitochondria were isolated from the right and left cerebral hemispheres (with subcortical areas), as previously described^115^. Cerebral tissue was weighed and transferred into a tube containing 1 ml of ice-cold isolation medium (IM), consisting of 225 mm mannitol, 75 mm sucrose, 20 mm MOPS, 1 mm EGTA, and 0.5 mm dithiothreitol, pH 7.4, and gently rinsed. The buffer was removed and IM containing Subtilisin A protease was added (1 ml/100 mg tissue), and the tissue was minced and homogenized with a with a glass/glass homogenizer for 10 min. Homogenate was further diluted 1:4 with IM, centrifuged at 2000 g for 4 min, the resultant supernatant passed through cheesecloth, the filtrate collected and centrifuged at 9000 g for 10 min. The resulting pellet was suspended in 6 ml of IM containing 0.02% digitonin, homogenized for 10 min, and centrifuged at 9000 g for 5 min. The resultant pellet was washed with 1 ml of IM and centrifuged again at 9000 g for 5 min. The final pellet was resuspended in 125 μl of IM/100 mg of tissue.

##### Mitochondrial oxygen consumption and reactive oxygen species production:

Mitochondrial respiration and H2O2 production were measured simultaneously using Oxygraph-O2K-Fluorescence LED2-Module (Oroboros Instruments, Innsbruck, Austria), as previously described^115, 116^. Oxygen consumption rate (OCR) and reactive oxygen species (ROS) production were measured in a 50 μl aliquot of isolated mitochondria suspension added to each 2 ml Oxygraph chamber and allowed to equilibrate. Mitochondrial respiration was measured devoid of substrates (state 1); in the presence of 10 mM glutamate, 2 mM malate, and 10 mM succinate (state 2); and 2.5 mM ADP (state 3). This was followed by addition of 2 μg/ml oligomycin to inhibit ATP synthase activity and induce state 4 respiration. Finally, 2.5 μM antimycin A was added to inhibit mitochondrial oxygen consumption and measure residual oxygen consumption. Mitochondrial H2O2 production was measured by continuous monitoring of Amplex Red oxidation (ThermoFisher Scientific, Waltham, MA, USA). Protein content from isolated mitochondria was determined using the DC Protein Assay (Bio-Rad Laboratories, Hercules, CA, USA). OCR and ROS emission were normalized per milligram of mitochondrial protein (reflective of mitochondrial protein quality) and per tissue wet weight (reflective of mitochondrial content).

##### Mitochondrial ATP production:

Mitochondrial ATP production was measured using an enzymatic system containing hexokinase and glucose-6-phosphate dehydrogenase to convert ATP to NADPH through sequential formation of glucose-6-phaospahate and 6-phosphoglucolactone using glucose and NADP+ as previously described^117^. A Fluorolog 3 (Horiba Scientific, Piscataway, NJ, USA) spectrofluorometer was used to continuously measure the autofluorescence of NADPH. Ten microliters of isolated mitochondria suspension was added to a quartz cuvette with 2 ml of buffer Z containing (in millimolars) 110 K-MES, 35 KCl, 1 EGTA, 5 K2HPO4, 3 MgCl2-6H2O, and 5 mg/ml bovine serum albumin (pH 7.4, 295 mOsm) and 2.5 mM D-Glucose. The same stepwise titration protocol was used to induce states 1, 2, 3, and 4 as previously described. OCR, ROS emission, and ATP production were normalized per milligram of mitochondrial protein (reflective of mitochondrial protein quality) and per tissue wet weight (reflective of mitochondrial content).

##### mtDNA quantification:

Total DNA was extracted using a DNA mini kit (Qiagen) and analyzed for mtDNA copy number using ND1 and ND4 genes, as previously described^75^. The abundance of each target gene was normalized to 28S ribosomal DNA and assayed in duplicate by quantitative PCR (qPCR) using a ViiA7 thermocycler (Applied Biosystems), using the appropriate primer sequences (28S: Fwd 5’- TGGGAATGCAGCCCAAAG – 3’, Rev 5’ – CCTTACGGTACTTGTTGACTATCG – 3’; Nd1: Fwd 5’ - AAGGAGAATCAGAATTAGTATCAGGGTT - 3’, Rev 5’ - TAGTACTCTGCTATAAAGAATAACGCGAAT - 3’; Nd4: Fwd 5’ - TCCAACTACGAACGGATCCA - 3’, Rev 5’ - AAGTGGGAAGACCATTTGAAGTC - 3’). qPCR was performed in 384 well clear plates with 20 μl reaction volume. Amplification conditions were 10 minutes at 60°C followed by 40 cycles of denaturing (95°C for 15 s) and annealing (60°C for 60 s). Each plate included a repeated control on the plate (intraassay control) and between plates (interassay control) along a no-template control, and 7-point relative standard curve spanning 4 log dilutions. Samples were amplified in triplicate. mtDNA copy number values were quantified by the 2ΔΔCt method^118^.

##### Immunoblotting:

Tissue samples were homogenized in RIPA buffer (EMD Millipore) with protease and phosphatase inhibitor cocktail (Thermo Fisher Scientific), resolved on SDS-PAGE gels, and blotted on nitrocellulose membranes, as previously described^75^. Membranes were blocked in TBST with 5% nonfat milk, and primary antibodies (Insulin receptor, Cell Signaling Technology, Cat. # 3025S, 1:1000; Beta-actin, LI-COR Biosciences, Cat. # 926–42212, 1:1000) were diluted in TBST with 5% BSA and applied to membranes overnight at 4°C. Appropriate secondary antibody (Anti-rabbit IgG, Cell Signaling Technology, Cat. # 7074,)1:5000; Anti-mouse IgG, Cell Signaling Technology, Cat. # 7076, 1:1000) was applied for 1 hour at room temperature in TBST with 5% nonfat milk, and proteins were detected by infrared fluorescence (LI-COR Odyssey).

##### Mitochondrial Enzyme Activities:

Maximal enzyme activities for citrate synthase (CS), cytochrome c oxidase (COX), and mitochondrial superoxide dismutase (SOD2) were determined spectrophotometrically in tissue homogenates (CS and COX) or isolated mitochondrial fractions (SOD2) from the cerebrum, hippocampus, and cortex. Tissue samples were homogenized in an ice-cold isolation buffer containing 100 mM KCl, 50 mM Tris-HCl (pH 7.4), 5 mM MgCl_2_, 1 mM EDTA, and 1 mM ATP. The homogenate was centrifuged at 800 × g for 10 minutes at 4°C to remove cellular debris. The resulting supernatant was further centrifuged at 10,000 × g for 15 minutes at 4°C to pellet mitochondria. The mitochondrial pellet was resuspended in the isolation buffer and kept on ice until analysis.

CS activity was measured by monitoring the rate of reduction of 5,5'-dithiobis-(2-nitrobenzoic acid) (DTNB) at 412 nm in a reaction mixture containing 100 mM Tris-HCl (pH 8.0), 0.1 mM DTNB, 0.3 mM acetyl-CoA, and 0.5 mM oxaloacetate. The reaction was initiated by adding oxaloacetate, and the increase in absorbance was recorded for 3 minutes at 25°C. COX activity was assessed by measuring the oxidation rate of reduced cytochrome c at 550 nm. The assay mixture comprised 10 mM potassium phosphate buffer (pH 7.0) and 50 μM reduced cytochrome c. The decrease in absorbance was monitored for 3 minutes at 25°C. CS and COX enzyme activities were normalized to protein content, which was determined using the Detergent Compatible (DC) Protein Assay kit (Bio-Rad, Hercules, CA, USA), following the manufacturer's instructions.

Mitochondrial superoxide dismutase (SOD2) activity was measured using a spectrophotometric assay based on the inhibition of xanthine oxidase–driven reduction of a tetrazolium salt by superoxide radicals (Superoxide Dismutase Assay Kit, Cayman Chemical, Cat# 706002). The assay quantifies the dismutation of superoxide radicals generated from the reaction of xanthine and xanthine oxidase, which reduces a chromogenic tetrazolium salt to formazan with peak absorbance at 450 nm. To selectively assess mitochondrial SOD2 activity, mitochondrial pellets were isolated as described above and incubated in the presence of 1 mM potassium cyanide (KCN) to inhibit Cu/ZnSOD (SOD1) activity. Absorbance was read at 450 nm, and the percentage inhibition of superoxide-induced formazan production was used to calculate SOD2 activity in U/mg protein. Protein concentrations were determined using the DC Protein Assay kit (Bio-Rad).

##### Y-maze Behavioral Testing:

Y-maze tests were used to assess spatial working memory and short-term spatial recognition memory in mice using two complementary paradigms: the Spontaneous Alternation Behavior (SAB) Working Memory Test and the Short-Term Memory (STM) Test. The maze consisted of three arms (labeled A, B, and C) of equal dimensions (40 cm long × 6.8 cm wide × 15.5 cm high) positioned at 120° angles from each other.

For the SAB Test, mice were placed at the end of one arm and allowed to freely explore the maze for 7 minutes, as previously described^119, 120^. During each 7-min period, the number of times that the tail of each animal fully entered each arm was counted for each arm, and then, the number of times each animal entered the arm one after another (in A, B, or C sequence) was also counted, which was assigned one point (actual alternation). Alternation behavior was defined as no overlap into all three arms and was calculated by the following formula: Rate of Spontaneous Alterations (%) = (Number of Alternations / Total Number of Arm Entries − 2) × 100.

For the STM Test, each arm of the Y-maze was visually differentiated by placing uniquely colored geometric shapes on the wall at the distal end of each arm to enhance spatial discrimination and a two-trial Y-maze task was conducted, as previously described^68, 121^. In the training phase, mice were allowed to explore two of the three arms (start and familiar arms) for 5 minutes while the third (novel) arm was blocked. After a 3-hour retention interval in their home cage, mice were reintroduced to the maze for a 5-minute test phase, during which all three arms were accessible. Time spent in each arm was quantified, and memory performance was expressed as the percentage of total arm exploration time spent in the novel arm Novel Arm Preference (%) = [Time in Novel Arm / (Time in Novel + Familiar Arms)] × 100. Increased preference for the novel arm during the test phase was interpreted as intact short-term spatial memory. The position of the novel arm was counterbalanced across animals to prevent spatial bias. The maze was thoroughly cleaned with 70% ethanol between trials to remove odor cues.

For the SAB Test and STM Test, the inside of the Y-maze was wiped with 70% ethanol between different animal trials. All tests were recorded by a technician blind to the genotype of the animals.

##### Statistical Analysis:

Unless otherwise stated, the results are expressed as means with individual data points shown to illustrate variability. Statistical analyses were performed using GraphPad Prism (version 8, GraphPad Software). To determine the significance of differences observed between Cre- and NIRKO groups in various outcomes, we employed two-sided unpaired Student’s t-tests. A significance level of p < 0.05 was considered statistically significant for all analyses. The exact value of n within the figures is indicated in the figure legends.

## Supplementary Files

This is a list of supplementary files associated with this preprint. Click to download.

- TableX18252025.pptx
- SupplementaryFigures8252025.pptx
- SUPPLEMENTALINFORMATION.docx
